# Genetic Variants Identified from Epilepsy of Unknown Etiology in Chinese Children by Targeted Exome Sequencing

**DOI:** 10.1038/srep40319

**Published:** 2017-01-11

**Authors:** Yimin Wang, Xiaonan Du, Rao Bin, Shanshan Yu, Zhezhi Xia, Guo Zheng, Jianmin Zhong, Yunjian Zhang, Yong-hui Jiang, Yi Wang

**Affiliations:** 1 Division of Neurology, Children’s Hospital of Fudan University, No. 399 Wanyuan Road, Shanghai, 201102, China; 2 BGI, Shenzhen, 518083, China; 3 Zhe Jiang Children’s Hospital, No. 3333 Binsheng Road, Hangzhou, Zhejiang, P.R. China; 4 Nan Jing Children’s Hospital, No. 72, Guangzhou Road, Nanjing, P.R. China; 5 Jiangxi Children’s Hospital, No.122, Yangming Road, Nanchang, P.R. China; 6 Division of Medical Genetics, Department of Pediatrics, Duke University School of Medicine, 905 S. LaSalle ST, Durham, NC USA; 7 University of Genomics and Genetics Program, Duke University, 905 S. LaSalle ST, Durham, NC USA; 8 Department of Neurobiology, Duke University School of Medicine, 905 S. LaSalle ST, Durham, NC USA; 9 Institute of Brain Science, Fudan University, 138,Yi Xue Yuan Rd, Shanghai, 200032, China

## Abstract

Genetic factors play a major role in the etiology of epilepsy disorders. Recent genomics studies using next generation sequencing (NGS) technique have identified a large number of genetic variants including copy number (CNV) and single nucleotide variant (SNV) in a small set of genes from individuals with epilepsy. These discoveries have contributed significantly to evaluate the etiology of epilepsy in clinic and lay the foundation to develop molecular specific treatment. However, the molecular basis for a majority of epilepsy patients remains elusive, and furthermore, most of these studies have been conducted in Caucasian children. Here we conducted a targeted exome-sequencing of 63 trios of Chinese epilepsy families using a custom-designed NGS panel that covers 412 known and candidate genes for epilepsy. We identified pathogenic and likely pathogenic variants in 15 of 63 (23.8%) families in known epilepsy genes including *SCN1A, CDKL5, STXBP1, CHD2, SCN3A, SCN9A, TSC2, MBD5, POLG* and *EFHC1.* More importantly, we identified likely pathologic variants in several novel candidate genes such as *GABRE, MYH1*, and *CLCN6*. Our results provide the evidence supporting the application of custom-designed NGS panel in clinic and indicate a conserved genetic susceptibility for epilepsy between Chinese and Caucasian children.

Genetic variations play an important role in the etiology of epilepsy disorders. Determining the pathogenicity of genetic variants in patients with epilepsy is critical for counseling families about the recurrent risk and developing the molecular specific treatment^1^,^2^,^3^,^4^. In the past decade, the number of genes implicated in epilepsy has been growing exponentially attributed to the advances of next generation sequencing (NGS) technology. These genetic discoveries have revolutionized the clinical practice to evaluate the molecular bases of epilepsy in epilepsy clinics^5^,^6^,^7^,^8^,^9^,^10^ and lay a foundation for the future development of precision medicine of epilepsy.

Despite these significant progresses, the molecular etiologies for the majority of epilepsy patients remain elusive. For the most of epilepsy patients with known genetic causes the genotype phenotype correlation has not yet been fully delineated. Furthermore, most epilepsy genetics and genomics studies have been conducted in Caucasian population and fewer in Chinese children. Whether the genetic susceptibility and mutation spectrum contributing to the epilepsy are shared between Caucasian and Chinese children has not been studied^11^,^12^.

Here we reported the results from a targeted exome-sequencing of 412 epilepsy candidate NGS gene panel in 63 Chinese trios with epilepsy of unclear etiology. We identified pathogenic and likely pathogenic variants in 15 of 63 (23.8%) families in the known epilepsy genes including *SCN1A, CDKL5, STXBP1, CHD2, SCN3A, SCN9A, TSC2, MBD5, POLG* and *EFHC1.* More importantly, we identified likely pathogenic variants in several novel candidate genes such as *GABRE, MYH1*, and *CLCN6*. Our results support the application of custom-designed NGS panel in epilepsy clinic and indicate that genetic susceptibility for epilepsy is highly conserved between Chinese and Caucasian population.

## Results

### Studying subjects

Epilepsy patients and their families were recruited from multiple Children’s Hospitals in China. The eligibility to participate the study was determined after medical record review by pediatric neurologists. Patients with acquired epilepsy such as hypoxic ischemic injury, infection of central nervous system, or other brain injury were excluded from the study. Clinical seizures were classified as following based on the recommendation of international league against epilepsy: **a**) infantile spasms (IS, n = 35), **b**) other types of epilepsies including, childhood absence epilepsy (CAE, n = 8; with 3 have family history), generalized epilepsy with febrile seizure plus (GEFS+, n = 2); febrile seizure (FS, n = 1); benign epilepsy with centro-temporal spikes with family history (BECT, n = 1), Dravet Syndrome (n = 2); Jeavons syndrome (n = 1); epileptic encephalopathy (EE, n = 3); epilepsy combined with autism (n = 3); epilepsy combined with intellectual disability (n = 5); photosensitive epilepsy (n = 1) and frontal lobe epilepsy (n = 1). Clinical information of the patients was summarized in Supplemental Table S1. Available family members were recruited when a co-segregation analysis for a given genetic variant was warranted. Patients had been followed for at least 1 year to determine their responses to anti-epilepsy drug (AED) treatment. Informed consent was obtained from parents of all subjects. The study was approved by the Intuitional Review Board of the Children’s Hospital of Fudan University [(2012) Protocol No. 185] and the study was conducted in accordance with relevant guidelines and regulations.

### Targeted exome sequencing

A custom-made NimbleGenSeqCap EZ Choice exon capture panel (Roche NimbleGen, Madison, USA) was designed specifically for this project. The coding exons, splicing sites and the immediately adjacent introns of 412 candidate genes were included in NGS panel (Supplemental Table S2). The selection of epilepsy candidate genes was based on curation and review of known epilepsy genes and functional candidates for epilepsy. These genes are categorized into the following groups based on the functional annotations: 1) ion channels (147 genes); 2) neurotransmitter and receptors (130 genes); 3) enzymes (31) and 4) other functional groups (104 genes). Among them, 85 genes were known to be causative genes for epilepsy based on the evidence curated in the Online Mendelian Inheritance in Man (OMIM).

**Figure 1:**
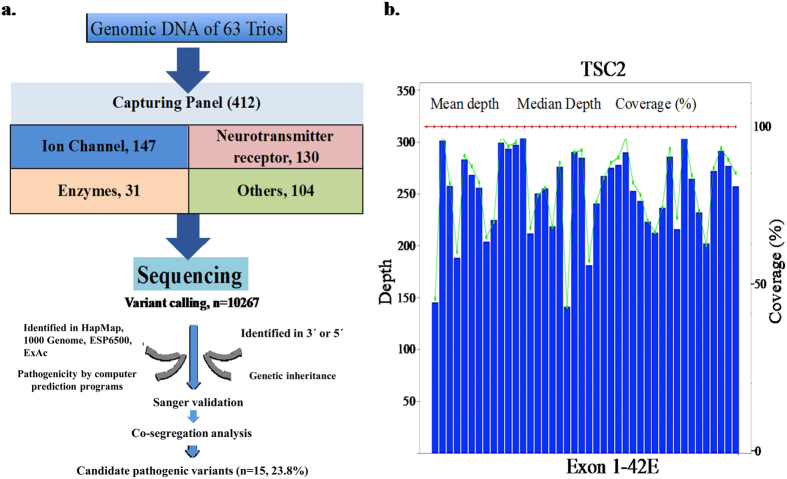
(**a**) A workflow of target exome sequencing and variant filtering protocol. (**b**) The average sequencing depth of coding exons of the representative gene *TSC2* from S4. ESP6500, NHLBI Exome Sequencing Project (ESP). ExAC, Exome Aggregation Consortium.

An average of 11.7 million paired-end reads with 90 bp in length were generated for each individual in trios. About 62.8% of total reads were mapped to the target regions and the average sequencing depth was 308-fold. A total of 10267 SNVs were called and then filtered by the 4-step process (Materials and methods). SNVs that passed the 4-step process were validated with Sanger sequencing (Fig. 1 and Supplemental Table S3). The pathogenicity of these variants are also assessed by multiple computational prediction programs for Pathogenicity and followed by careful annotation based on the standards and guidelines recommended recently by American College of Medical Genetics^13^ (Materials and Methods). We classified these variants to the following categories: pathogenic, likely pathogenic, uncertain significance, likely benign, and benign. Through this filtering process, we identified 24 SNVs [2 families (S86 and D1422) have compound heterozygotes and 2 families (D1339 and D1433) have more than 1 SNV] from 16 patients that are pathogenic or likely pathogenic (Table 1).

### Pathogenic variants

**Figure 2:**
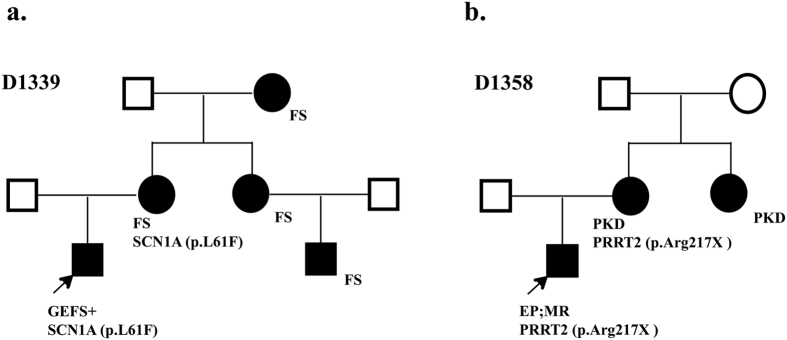
The recurrent mutations or novel variants familial cases. (**a)** A recurrent pathogenic mutation in *SCN1A* (c.181 C>T, p.leu61Phe) in family D1339. (**b)** A recurrent pathogenic mutation in *PRRT2* (c.649 C>T, p.Arg217X) in family D1358.

**Figure 3:**
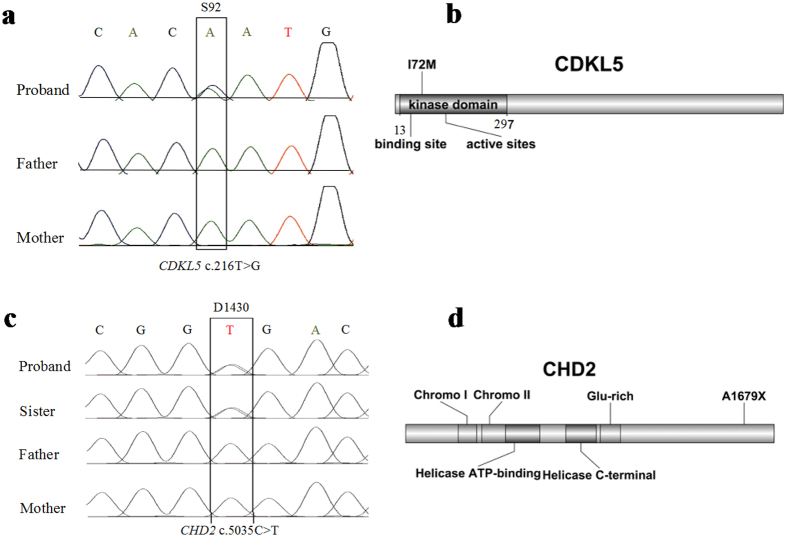
Examples of Sanger sequencing validation and the position of sequence variants in the corresponding proteins for *CDKL5* and *CHD2*.

We identified 8 pathogenic variants in 8 families. Six families have single variant and 2 families have additional variants (D1339 and D1433). These variants are either recurrent mutations or novel but deleterious variants in known epilepsy genes. We identified 2 recurrent mutations (c.311 C>T, p.Ala104Val and c.181 C>T, p.Leu61Phe) in *SCN1A* gene (http://www.ncbi.nlm.nih.gov/clinvar), a known epilepsy gene for Dravet syndrome (DS), generalized epilepsy with febrile seizures plus (GEFS+) and epileptic encephalopathy^14^,^15^,^16^. In D1353 family, a *de novo* but recurrent mutation of c.311 C>T was found in a boy with Dravet syndrome, intractable seizure, significant regression of intellectual ability, and autistic behaviors. A recurrent mutation of c.181 C>T was detected in a boy (D1339) with a diagnosis of GEFS+. He inherited this mutation from his mother who also has history of febrile seizure but no other significant clinical problem. His aunt, cousin and maternal grandmother also had history of febrile seizure but they were not available for the genetic test (Fig. 2a). In addition, a novel and *de novo* SNV of c.2948delT (p.V983Afs*2) in *SCN1A* was detected in a boy with intractable complex partial seizures and epileptic encephalopathy, and history of status epilepticus. Recurrent mutations were also found in *TSC2* (c.2197 C>G, p.Leu733Val) and *PRRT2* (c.649 C>T, p.Arg217X) in individual family S4 and D1358, respectively^17^,^18^,^19^. A 9-years-old boy carrying a *de novo* variant of c.2197 C>G in *TSC2* gene was suspected to have a clinical diagnosis of TSC at infant age because of clinical presentation of infantile spasms. However, the clinical diagnosis of TSC could not be established because of lacking other features to meet the major criteria of TSC diagnosis. The brain imaging was negative at the first presentation but the sub-ependymal nodules were seen at age of 9 years and the diagnosis of TSC was then established. He also has significant intellectual disability (ID). His infantile spasms were well controlled by the combined therapy of Valproate and Topiramate. A nonsense variant of c.649 C>T (p.Arg217X) in *PRRT2* has been previously reported in a Chinese family with sporadic paroxysmal kinesigenic dyskinesia (PKD)^20^. In family of D1358, the same mutation c.649 C>T is inherited from mother who has a diagnosis of mild paroxysmal dyskinesia (Fig. 2b). Neither of them had seizure disorder. *CDKL5* and *STXBP1* are known to implicate in early infantile epileptic encephalopathy (EIEE) or Ohtahara syndrome^21^,^22^. We identified *de novo* variants of c. 216 T>G (p.Ile72Met) of *CDKL5* (Fig. 3a,b) and c.54delG in *STXBP1* in the proband of S92 and S163 families respectively. A *de novo* nonsense mutation of c.5035 C>T (p.Arg1679X) in *CHD2* was found in monozygotic twin girls with generalized epilepsy in the family of D1430 (Fig. 3c,d). Both girls developed seizures at age 3 year that is photosensitive and shared nearly identical manifestations including intellectual disability, abnormal EEG, and tonic-clonic seizure. Most patients carrying *CHD2* mutation in literature developed seizure before 3 years of age^23^,^24^,^25^,^26^,^27^, suggesting this gene might be critical for early brain development and function^28^. The nonsense mutation in our patients is located in the C terminus of CHD2 protein but it is not known whether the nonsense mediated decay mechanism is involved. Two nearby frame-shift mutations (p.Arg1644Lysfs and p.Lys1419Argfs) have been reported in patients with myoclonic-astatic epilepsy^26^,^27^. Both cases had early onset epileptic seizures. The development is normal before seizure onset but severe intellectual disabilities developed after seizure onset. In one case, patient also responded well to valproate (VPA) treatment.

Interestingly, two families (D1433 and D1339) were found to have more than one variant in known epilepsy genes. In D1433 family, in addition to the causal mutation of c.2948delT in *SCN1A*, we also identified a novel variant of c.4214 G>A (p.Arg1405Gln) in *CACNA1H*, a gene that encodes an alpha-1 subunit of calcium channel. Variants in *CACNA1H* gene have been suggested to associate with epilepsy in two studies but the evidence is relatively weak^29^,^30^. In family D1339, two additional variants were found in *KCNQ5* (c.7 C>T, p.Arg3Cys) and *UGT1A4* (c.1378 G>A, p.Val460Met) respectively in proband who also carries a known recurrent pathogenic variant (c.181 C>T) in *SCN1A* gene. *KCNQ5* encodes a subfamily member of voltage-gated potassium channel. There is no report in literature that support the role of *KCNQ5* in epilepsy but mutations in other voltage-gated potassium channel subfamily members such as *KCNQ2* have been strongly implicated in epilepsy^31^. *UGT1A4* encodes the UDP glycosyltransferase that is likely to contribute to the protein glycosylation. Similarly, there is no evidence supporting a role of *UGT1A* in the susceptibility of epilepsy.

### Likely pathogenic variants

**Figure 4:**
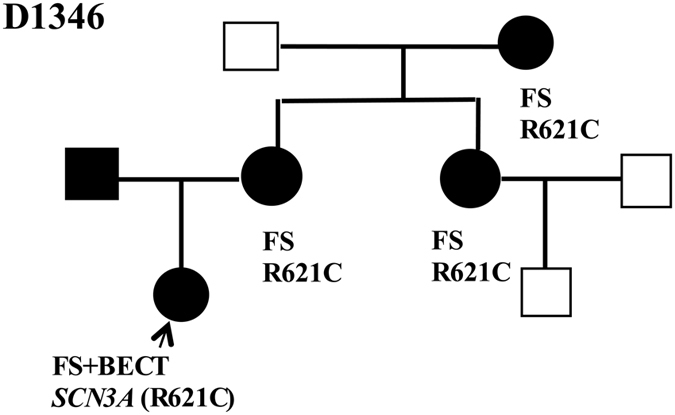
A novel likely pathogenic SNP (c.1861 C>T, p.Arg621Cys) in SCN3A. FS: febrile seizures; GEFS+: generalized epilepsy with FS plus; PKD paroxysmal kinesigenic dyskinesia.

**Figure 5:**
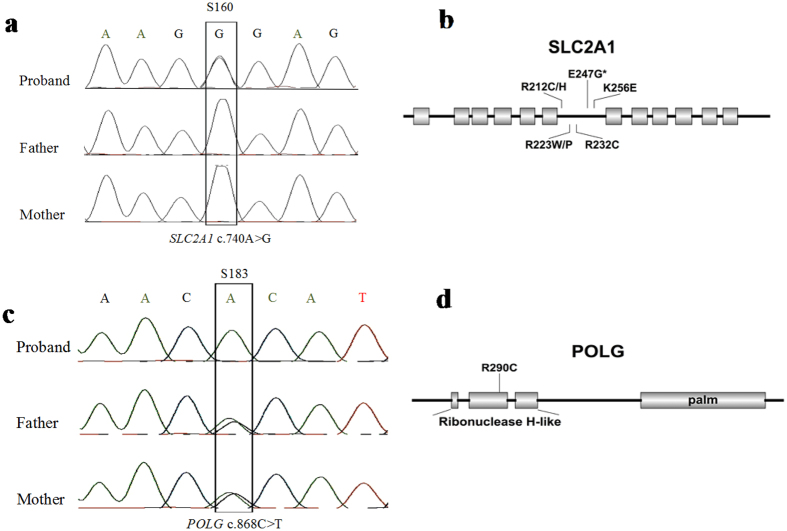
Examples of Sanger sequencing validation and the position of sequence variants in the corresponding proteins for *SLC2A1*, and *PLOG*.

We identified 8 variants in 7 families that are likely pathogenic. Collectively, variants in known or novel genes found in these families are very suggestive for the pathogenicity but the evidence to support this classification varies. These variants are rare, *de novo* in most of case, and predicted to be damaging for the protein function from multiple computational programs. In family S23, an 8-year-old boy with infantile spasms is homozygotes for a variant of c.1355 G>T (p.Arg452Leu) in *GABRE* gene that is inherited from healthy parents. *GABRE* is mapped to the chromosome Xq28 and encodes a gamma-aminobutyric acid (GABA) A receptor epsilon polypeptide that involves in the GABAergic neurotransmission of the mammalian central nervous system^32^,^33^. Several other members of GABA receptor but not *GABRE* have been implicated in human epilepsy in human^34^,^35^. Variants of *SCN9A* in heterozygote have been reported in patients of generalized epilepsy with febrile seizure plus (GEFS+), familiar febrile seizure, and other non-epilepsy related phenotypes^36^. Mutations in *SCN9A* that follow the recessive inheritance have been identified in family with pain insensitivity^37^. In family D1422, we identified a compound heterozygotes of c.3719 A>G (p.Lys1240Arg) and c.121 G>C (p.Asp41His) in a proband with CAE. In a family D1346 that has a strong family history of febrile seizure, we detected a missense variant of (c.1861 C>T, p.R621C) in *SCN3A* gene in multiple family members (Fig. 4). The variant is inherited from his mother and maternal grandmother as well as from maternal aunt who have history of febrile seizure. Interestingly, the proband’s father who was also affected by febrile seizure during infant and childhood but does not carry the same *SCN3A* variant. This may suggest a different risk gene that is not in our NGS panel is responsible for his presentations. Similarly, in family S86, compound heterozygous mutation of *MYH1* gene (c.3947 T>C, p.Ile1316Thr and c.92 C>T, p.Pro31Leu) was found in a proband with infantile spasms. In family S160, a *de novo* missense variant of c.740 A>G (p.E247G) in *SLC2A1* gene that encodes glucose transporter (GLUT1) was found in a girl with infantile spasms. Deficiency of glucose transporter *SLC2A1* is implicated in several neurological disorders. Historically, homozygous mutation in *SLC2A1* that encodes glucose transporter GLUT1, is the cause for autosomal recessive glucose transporter defect disorder in which the hypoglycemia induced seizure is the major clinical feature. Heterozygous mutations following an autosomal dominant inheritance have also been implicated in dystonia 9 (DYT9) and idiopathic generalized epilepsy (EIG12) respectively^38^,^39^,^40^. The p.E247G variant found in our patient is located in the middle of cytoplasmic region of GLUT1 and several known disease causing mutations close to this site for DYT9 and EIG12 have been reported (Fig. 5a,b)^39^,^40^,^41^,^42^. In family S183, a novel homozygous variant of c.868 C>T (p.Arg290Cys) in the second ribonuclease H-like domain of *POLG* (Fig. 5c,d) was found in a 2-year-old boy with infantile spasms. Mutations of both autosomal recessive or dominant inheritance in *POLG* gene have been implicated in number of diseases in which neurological and seizure presentations are common. In family D1435, a *de novo* missense variant of *CLCN6* (c.533 A>C, p.Glu178Ala) which encode voltage gated chloride channel, was detected in a boy with infantile spasms. The Glu178Ala variant is located in one of the transmembrane helix that was likely involved in ion transportation. The role of *CLCN6* in epilepsy has been suggested but not confirmed in two other studies^43^,^44^. Two SNVs in *CLCN6* have been identified in patients with benign partial epilepsies in infancy (BPEI) or benign familial infantile epilepsy (BFIE) but the causal role has not been established^43^,^44^. Because of these observations, we classified this *de novo* missense variant of *CLCN6* (c.533 A>C) as likely pathogenic but with weak evidence.

### Variant of unknown significance(VUS)

Six variants are classified as VUS. In family D1383, we detected rare SNVs in four different genes including CYP2C9 (c.445 G>A, pAla149Thr), *EFHC1* (c.1906 C>T), p.Arg636Cys), *KCNN4* (c.766Gly>Ala, p.Val256Met), and RYR2 (c.7502, p.Ile2351Thr) in a 6-year-old boy diagnosed with CAE. All four variants were inherited from his father who was also diagnosed with *CAE. EFHC1, CYP2C9*, and *RYR2* genes are known to be implicated in epilepsy^45^,^46^,^47^. *KCNN4* encodes a member of calcium-activated potassium channels. While there is no direct evidence supporting the involvement of *KCNN4*, other members of potassium channel, such as *KCNT1*, have been strongly implicated in epilepsy^48^,^49^,^50^. *MDB5* gene has been strongly implicated in epilepsy and autism spectrum disorders^51^,^52^. We identified two novel but inherited missense variants: c.1885 A>G (p.Asn629Asp) in D1471 and c.365 C>T (p.Ser122Phe) in D1426, in MBD5 gene in two unrelated families respectively (Table 1). While multiple paternal family members of proband in D1426 have clinical seizure, the SNV of c.365 C>T was not segregated with seizure in family. Thus, the clinical relevance of this variant is not clear (Supplementary Figure S1). Similarly, the c.1885 A>G variant in *MBD5* in a boy (D1471) with Lennox-Gastaut Syndrome (LGS) was inherited from his mother who was reported to have possible febrile seizure during childhood.

## Discussion

Using a customer designed target exome sequencing platform, we have conducted the first NGS for 63 trios of Chinese families with childhood epilepsy of unknown etiology. Our targeting exome panel includes 85 known epilepsy genes and 320 candidate genes for epilepsy. We were able to determine the definitive causes in 8 families because the pathogenic or causal variants were identified in known epilepsy genes including *SCN1A, CDKL5, STXBP1, CHD2, SCN3A, SCN9A, TSC2, MBD5, POLG* and *EFHC1*. Mutations in these genes have been commonly identified in Caucasian children with epilepsy disorders. Notably, several mutations are recurrent and have been reported in Caucasian population with similar clinical presentations. These findings support clinical usage of the NGS panel in epilepsy clinic to determine the molecular bases underlying epilepsy. Our findings also support that the genetic susceptibility for epilepsy is shared among different races. While the clinical presentations for these patients carrying the recurrent mutations are similar to the Caucasian children based on the review of the data available in the reports^14^,^15^,^16^,^26^,^27^. It would be interesting in future study to compare them systematically in parallel with larger sample size and determine whether a significant modifier effect may be present in different genetic background. In other 7 families, we detected the variants in known or novel epilepsy candidate genes including *GABRE, MYH1*, and *CLCN6.* We classified these variant as likely pathogenic variants but the evidence supports these classifications varies. The roles of these genes in epilepsy have not been reported in literature and additional studies are clearly warranted to support the pathogenicity. For example, a compound heterozygotes were found in *MYH1* gene that encodes a myosin heavy chain and presumably implicate in the function of skeletal muscle. *MYH1* encodes a myosin heavy chain, a ubiquitous actin-based motor protein that converts the chemical energy derived from hydrolysis of ATP into mechanical force. However, little is known whether this gene is also expressed in brain and may have a different function in brain. Additional functional studies are warranted to determine whether these variants are causal and how dysfunction of *MYH1* may cause infantile spasms mechanistically

Interestingly, we detected more than one rare and *de novo* sequence variants in several families. For example, in the families of D1339 and D1433, the pathogenic variants are identified in *SCN1A* in probands. However, we also detected additional variants in *KCNQ5* and *UGT1A* genes in proband of D1339 and *CANCA1H* in D1433 family. *KCNQ5* encodes the sub-family of potassium channel and *CANCA1H* encodes a subunit of calcium channel. Currently, there is no evidence supporting a causal role of these genes in epilepsy. However, mutations in other family members of potassium and calcium channels are strongly implicated in epilepsy or other neurological disorders^48^,^49^,^50^. The interesting question is whether the variants in these genes may function as a modifier to contribute to the clinical phenotypes caused by the mutations in *SCN1A*. The extreme variable expressivity is well documented for individual with *SCN1A* mutation^14^,^15^,^16^. However, little is known about the underlying molecular mechanism contributing to the variable expressivity associated with *SCN1A* mutations. The additional variants found in individuals with *SCN1A* pathogenic mutation will allow us to formulate the hypothesis and dissect the mechanism.

In family of D1383, rare variants inherited paternally are found in 4 genes (*CYP2C9. EFHC1. KCNN4*, and *RYR2*) in proband with CAE and the father also has seizure disorder. While the individual variant is likely fall in the category of VUS, the combination may suggest a possibility of an oligogenic model of genetic susceptibility for certain type of epilepsy, more evidence from human and functional studies is required.

Recently, targeted capture using NGS panels have been used in both research and clinical molecular genetics laboratories to understand the molecular etiology of epilepsy disorders^53^. The NGS panels used in research studies vary in gene composition, sample size, inclusion criteria, and sequence analytic protocol. An average of ~30% positive yield has been reported in different reports but a direct compassion for these data will not be informative^8^,^11^,^26^,^54^,^55^,^56^. In this study for Chinese epilepsy children using this approach, we identified pathogenic and likely pathogenic candidate variants in 23.8% of patients^11^. Compared to other studies in literature, our panel contains significant more genes in NGS panel. However, we noted that the positive detection rate for causal variants is not significantly increased. Our finding may suggest a challenge of choosing candidate genes genome wide based on the functional prediction and *in silico* analysis.

In summary, our study supported the use of NGS panel as an effective tool to detect the genetic cause for epilepsy of unknown etiology. Our study generated a list of novel sequence variants that will enrich the spectrum of genetic variants implicated in epilepsy. Our findings provide several potential insights or a list of sequence variants for further functional investigation to understand the complex molecular mechanism underlying the epilepsy disorders.

## Material and Methods

### Targeted exon capture and sequencing

Genomic DNA was extracted from blood sample from each participant using the QIAamp DNA Blood Mini Kit (Qiagen, Hilden, Germany) according to the manufacturer’s instructions. The genomic DNA was fragmented to the size of 200bp-250bp by CovarisLE220 (Massachusetts, USA). Then pair-end DNA libraries were prepared following Illumina paired-end protocols. Adapter-ligated libraries were amplified and indexed via PCR. All the libraries were hybridized to NimbleGenSeqCap EZ Choice Library to enrich target region exons. Pooled libraries were subjected to massively parallel sequencing by 90 pair-end reads on a Hiseq2000 sequencer (Illumina, San Diego, California).

### Sequence alignment and variant calling

Raw data of fastq format after image analysis and base calling was processed using the Illumina Pipeline. Clean reads were generated for further analysis by removing the adapters and the low quality reads (contained more than 10% Ns in the read length, 50% reads with a quality value less than 10). Filtered reads were aligned to the human reference genome (Hg19, NCBI Build 37.5) using the BWA Multi-Vision software package (version 0.7.10). Duplicate reads were marked by Picard toolkit (version 1.95). Single nucleotide variant (SNV) and indel (insertion or deletion) calling was done by the Genome Analysis Tool Kit (version 3.1.1) after local realignment and quality recalibration. SIFT (Human_db_37_ensembl_63, released Auguest, 2011), PolyPhen-2 (version 2.2.2, released Feb, 2012), LRT (released November, 2009), MutationTaster (data retrieved by 2013), MutationAssessor (release 2) and FATHMM (version 2.3) were applied to predict the effects of mutations on protein structure and function. Finally, multiple sequence alignment was performed using Blast (http://www.uniprot.org/blast/) to analyze the degree of conservation of the predicted amino acid substitutions.

### Variant filtering and annotation

In total, 10267 variants of affected person were generated through BWA, Picard and GATK pipeline, which were then processed by following 4 step process in order to select the candidate pathogenic mutations: (1) The allele frequencies of the mutation in dbSNP, HapMap database, 1000 Genomes Project should all be ‘0’. The allele frequencies of remained mutation in ESP6500 AF and ExAC East Asian AF should be less than 0.1% (2) Mutations in coding regions (missense, nonsense, splice-variant, coding indels and frameshift) which was predicted to be deleterious were retained (3) Co-segregation analysis was carried out under the *de novo*, autosomal dominant, and autosomal recessive model, based on the family history. Those that did not follow the inheriting pattern were excluded. (4) Missense variants that were retained should be predicted as “Damaging” by at least one of the six software previously introduced.

### Sequence variant classification for the pathogenicity

The pathogenicity of sequence variant is classified to the following categories: 1) pathogenic, 2) likely pathogenic, 3) uncertain significance, 4) likely benign, and 5) benign. We followed the principle of standards and guidelines recommended by ACMG (American College of Medical Genetics) in recent publication^13^.

### Sanger sequencing

Primers were designed to span at least 100 bp upstream and downstream of the variant identified by Hiseq2000. Mutations that passed the rare variant segregation test were confirmed by Sanger sequencing. PCR amplification was conducted in an ABI 9700 Thermal Cycler using standard conditions. And products were purified using a Universal DNA Purification Kit (Tiangen) and directly sequenced on an ABI PRISM 3730 automated sequencer (Applied Biosystems). Sequencing reads were compared using Chromas software. The DNA template and PCR conditions were listed in Supplemental Table S3.

## Additional Information

**How to cite this article**: Wang, Y. *et al*. Genetic Variants Identified from Epilepsy of Unknown Etiology in Chinese Children by Targeted Exome Sequencing. *Sci. Rep.* **7**, 40319; doi: 10.1038/srep40319 (2017).

**Publisher's note:** Springer Nature remains neutral with regard to jurisdictional claims in published maps and institutional affiliations.

## Supplementary Material

Supplementary Figure S1

Supplementary Table S1

Supplementary Table S2

Supplementary Table S3

## Figures and Tables

**Table 1:** Candidate variants and the feature of clinical profiles of studying patients.

Proband ID	Gender/ Onset age	Variants	Inheritance	Diagnosis	Seizure type	Development	EEG/VEEG	Brain Imaging	Prognosis	Remark
**Pathogenic variants**										
D1353	M/6 m	SCN1A (c.311 C>T, p.Ala104Val)	*de novo*	Dravet syndrome	Febrile seizure and myoclonic seizure	ID, ASD Features	EEG: Epileptic discharge at 6 m; slow spike and weave at 1y; sharp wave, sharp and slow wave complex at 3y	Normal (MRI)	Intractable to OXC, LEV, VPA and vagus nerve stimulation; seizure free for 2 months to VPA+CLA+PB	Epilepsy family history
D1339	M/8 m	SCN1A (c.181 C>T, p.Leu61Phe)	M	GEFS+	Febrile seizures	Normal	EEG: Sharp waves and slow wave complex	Normal (MRI)	Seizure free for 2.5y with VAP+LEV	FS family history (mother, maternal aunt and grandma were diagnosed with FS)
		KCNQ5 (c.7 C>T, p.Arg3Cys)	*de novo*							
		UGT1A4/6 (c.1378 G>A, p.Val460Met)	*de novo*							
D1433	M/5 m	SCN1A (c.2948delT, p.Val983Alafs*2)	*de novo*	Dravet Syndrome	Complex partial seizures, secondarily generalized status epilepticus	ID	VEEG: Mass of high δ and ϴ waves at 14 m; sharp waves, sharp and slow wave complex at 2.5y	Enlarged extracerebral gap (MRI, 15 m)	Intractable to VPA, OXC, CLZ, and LEV	
		*CACNA1H* (c.4214 G>A, p.Arg1405Gln)								
S92	F/1.5 m	CDKL5 (c.216 T>G, p.Ile72Met)	*de novo*	IS	Spasms	GDD	EEG: Hyperarrhythmia	Cerebral dysplasia (MRI, 3 m)	Intractable to VPA+TPM	
S4	M/4 m	TSC2 (c.2197 C>G, p.Leu733Val)	*de novo*	IS	Spasms change to myoclonic seizures	ID/GDD	EEG: Hyperarrhythmia	Enlarged extracerebral gap (CT, 4 m); subependymal nodules (CT, 9y)	Intractable to VPA+TPM+CLZ+LEV	4 skin Hypomelanotic macules
D1358	M/5 m	PRRT2 (c.649 C>T, p.Arg217x)	M	EP	Tonic-Clonic seizures	ID	EEG: Sharp and slow wave complex	Bilateral temporal angle hyperplasia, bilateral parietal lobe abnormal signal (MRI; 3y)	Seizure free for 2y to VPA; after recurrence, seizure free for 1y to VPA+OXC	Mother and maternal aunt diagnosed with PKD
S163	F/6 m	STXBP1(c.54delG, p.Val18fs*1)	*de novo*	IS	Spasms in cluster; myoclonic seizures; Tonic-Clonic seizures	ID	EEG: Hypsarrhythmia	Enlarged extracerebral gap (MRI; 2y)	Seizure free for 1.5 years to VPA+TPM	
D1430	F/3y	CHD2(c.5035 C>T, p.Arg1679x)	*de novo*	EP	Tonic-Clonic seizures	ID	EEG: Spike and sharp wave	Normal (MRI)	Seizure free for 1 year to VPA	The same mutation exists in his monozygotic sister with similar clinical feature
**Likely pathogenic**										
D1346	F/5y	SCN3A (c.1861 C>T, p.Arg621Cys)	M	BECTs	Tonic-Clonic seizures	Normal	EEG: Centro-temporal spikes	Normal (MRI)	Seizure free for 2 years to OXC	
D1422	F/5y	SCN9A (c.121 G>C, p.Asp41His) SCN9A (c.3719 A>G, p.Lys1240Arg)	M	Jeavons syndrome	Absence, eyelid myoclonia	Normal	EEG: 3 Hz spike-wave	Normal (MRI)	Seizure free to VPA	
S23	M/6 m	GABRE (c.1355 G>T, p.Arg452Leu)	M	IS	Spasms in cluster	GDD	EEG: Hyperarrhythmia at 7 m; multiple spike and slow wave complex at 1y	Myelin development delay (MRI, 7 m); brain dysplasia (MRI, 22 m)	Seizure reduction by >75% to VPA+TPM +CLZ	
S86	F/4 m	MYH1 (c.3947 T>C, p.Ile1316Thr) MYH1 (c.92 C>T, p.Pro31Leu)	F/M	IS	Spasms in cluster	GDD	EEG: Hyperarrhythmia	Normal (MRI)	Intractable to VPA+TPM+CLZ and 6-months ketogenic diet	
S160	F/3 m	SLC2A1 (c.740 A>G, p.Glu247Gly)	*de novo*	IS	Spasms in cluster; myoclonic seizures; Tonic-Clonic seizures	ID	EEG: Atypical hyper arrhythmia	Normal (CT)	Seizure reduction by75% to VPA+TPM	Skin cafe-au-lait spots
S183	M/5 m	POLG (c.868 C>T, p.Arg290Cys)	*de novo*	IS	Spasms in cluster; myoclonic seizures; Tonic-Clonic seizures	GDD	EEG: Hyperarrhythmia	Normal (MRI)	Seizure free for one year to VPA+TPM	Unable to walk and self-feed until 4-years old; cannot talk and showed poor eye contact.
D1435	M/5 m	CLCN6 (c.533 A>C, p.Glu178Ala)	*de novo*	IS	Spasms in cluster change to Tonic-Clonic and myclonic seizure	ID	EEG: Hyperarrhythmia	Enlarged subarachnoidale (MRI)	Seizure free for two month to ACTH, and then recurred. Intractable to VPA+TPM+NZP. Spasms stopped at 2.5y without AED and then recurred at 6y	microcephaly (<3 SD)
**Variant of Unknown significance (VUS)**										
D1383	M/6y	CYP2C9 (c.445 G>A, p.Ala149Thr)	M	CAE	Absence	Normal	EEG: 3 Hz spike-wave	Normal (MRI)	Seizure free for 2 years to VPA	Father has CAE
		EFHC1 (c.1906 C>T, p.Arg636Cys)								
		KCNN4 (c.766 G>A, p.Val256Met)								
		*RYR2(*c.7052 T, p.Ile2351Thr)								
D1426	F/15 m	MBD5 (c.365 C>T, p.Ser122Phe)	F	EP	Tonic-Clonic seizures	ID	EEG: No epileptic discharge	Bilateral ventricle and three ventricle enlargement, periventricular white matter less (MRI)	About two times of epileptic seizures annually without AED drugs	Congenital heart disease(patent ductus arteriosus, atrialseptal defect), craniofacial abnormalities
D1471	M/1.5y	MBD5 (c.1885 A>G, p.Asn629Asp)	M	EIEE	Tonic-clonic seizures with fever and status epilepticus; transformed to myoclonic seizures and spasms	GDD	VEEG: Spike wave, sharp wave at left frontal lobe, frontal lobe	Absence of septum pellucidum, enlarged bilateral ventricle, finer bilateral artery (MRI)	Intractable to VPA+TPM; response to ACTH initially but relapsed 6 months later	developmental regression and loss of language after 2.5y. Partial recovery was observed after ACTH

Note: M, F in the column of Gender/Onset represent male and female respectively. In all part of this table, m and y following number represent month and year. M, F in the column of inheritance represent mother and father respectively. Generalized epilepsy with febrile seizures plus (GEFS+), Infantile spasm (IS), Early Infantile Epileptic encephalopathy (EIEE), and Epilepsy (EP). Intelligence disability (ID), Autism spectrum disorders (ASD), and Globel development delay (GDD). Electroencephalograph (EEG), Video electroencephalograph (VEEG). Magnetic Resonance Imaging (MRI), Computed Tomography (CT). Oxcarbazepine (OXC), Valproate (VPA), Levetiracetam (LEV), Clonazepam (CLZ), PB (Phenobarbital), Topamax (TPM), and ACTH (adreno-cortico-tropic-hormone). Febrile seizure (FS), Paroxysmal Kinesigenit Dyskinesia (PKD).
