# Corrigendum: Genetic Variants Identified from Epilepsy of Unknown Etiology in Chinese Children by Targeted Exome Sequencing

**DOI:** 10.1038/srep46520

**Published:** 2017-05-04

**Authors:** Yimin Wang, Xiaonan Du, Rao Bin, Shanshan Yu, Zhezhi Xia, Guo Zheng, Jianmin Zhong, Yunjian Zhang, Yong-hui Jiang, Yi Wang

Scientific Reports 7: Article number: 4031910.1038/srep40319; published online: 01 11 2017; updated: 05 04 2017

This Article contains a typographical error in the Results section under subheading “Targeted exome sequencing”, where:

“Through this filtering process, we identified 24 SNVs [2 families (S86 and D1422) have compound heterozygotes and 2 families (D1339 and D1433) have more than 1 SNV] from 16 patients that are pathogenic or likely pathogenic (Table 1)”.

should read:

“Through this filtering process, we identified 24 SNVs [2 families (S86 and D1422) have compound heterozygotes and 2 families (D1339 and D1433) have more than 1 SNV] from 15 patients that are pathogenic or likely pathogenic (Table 1)”.

In addition, the Supplementary Information File originally published with this Article contained errors in Supplementary Table S1. These errors have now been corrected in the Supplementary Information that accompanies the Article.

