# Supplementary material for: Genetic Variants Identified from Epilepsy of Unknown Etiology in Chinese Children by Targeted Exome Sequencing
Source: Sci Rep. 2017 Jan 11;7:40319. doi: 10.1038/srep40319 (PMC5225856; doi:10.1038/srep40319)
Supplement: Supplementary Figure S1 [file srep40319-s1.pdf]

Genetic Variants Identified from Epilepsy of Unknown Etiology in Chinese Children by Targeted Exome Sequencing

Yimin Wang[1#], Xiaonan Du[1#], Rao Bin[2], Shanshan Yu[2], Zhezhi Xia[6], Guo Zheng[7], Jianmin Zhong[8], Yunjian Zhang[1], Yong-hui Jiang[3,4,5]*, Yi Wang[1,9]*

1. Division of Neurology, Children's Hospital of Fudan University, No. 399 Wanyuan Road, Shanghai, 201102, China
2. BGI, Shenzhen, 518083, China
3. Division of Medical Genetics, Department of Pediatrics, Duke University School of Medicine, 905 S. LaSalle ST, Durham, NC USA
4. University of Genomics and Genetics Program, Department of Pediatrics, Duke University, 905 S. LaSalle ST, Durham, NC USA
5. Department of Neurobiology, Duke University School of Medicine, 905 S. LaSalle ST, Durham, NC USA
6. Zhe Jiang Children's Hospital, No. 3333 Binsheng Road, Hangzhou, Zhejiang, P.R. China
7. Nan Jing Children's Hospital, No.72, Guangzhou Road, Nanjing, P.R.China
8. Jiangxi Children's Hospital, No.122, Yangming Road, Nanchang, P.R. China
9. Institute of Brain Science, Fudan University, 138,Yi Xue Yuan Rd, Shanghai, 200032, China

#; co-first author

***Correspondence:**

Wang Yi, MD, Ph.D

Division of Neurology,

Children’s Hospital of Fudan University

No 399 Wan Yuan Road, Shanghai City 201102 P.R. China

Tel: 86-21-64931905

Email: yiwang@shmu.edu.cn

Yong-hui Jiang, MD, PhD

Department of Pediatric

Duke University School of Medicine

Durham NC USA 27710

Tel:919-681-2789

Email: yong-hui.jiang@duke.edu

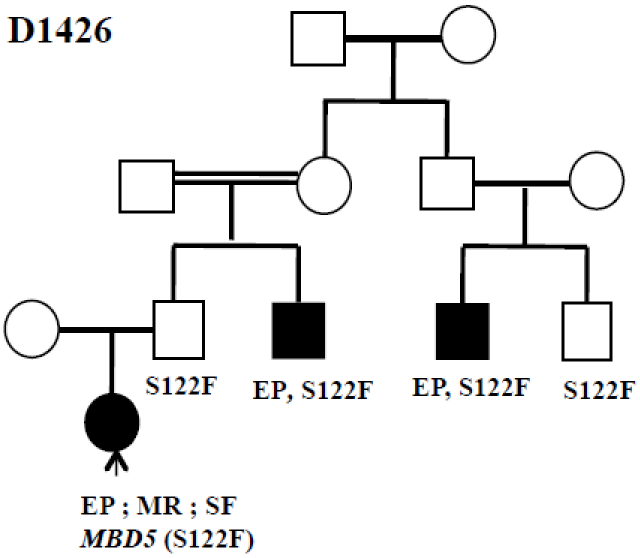

Supplementary Figure S1: A novel SNP of VUS identified in o family D1426 (c.365 C>T, p.Ser122Phe) in MBD. EP : Epilepsy.
